# The Efficacy of Intravitreal Conbercept for Chronic Central Serous Chorioretinopathy

**DOI:** 10.1155/2019/7409426

**Published:** 2019-05-07

**Authors:** Jianbo Mao, Caiyun Zhang, Chenyi Liu, Lijun Shen, Jimeng Lao, Yirun Shao, Yiqi Chen, Jiwei Tao

**Affiliations:** ^1^Eye Hospital of Wenzhou Medical University, Wenzhou, Zhejiang, China; ^2^Chicago College of Optometry, Midwestern University, Downers Grove, IL, USA

## Abstract

**Purpose:**

To evaluate the efficacy and safety of conbercept for patients with chronic central serous chorioretinopathy (CSC).

**Methods:**

A retrospective clinical study. Thirty-one patients (35 eyes) with chronic CSC were given intravitreal injections of conbercept and followed up for at least 6 months. Observed indicators included best-corrected visual acuity (BCVA), central macular thickness (CMT), and resolution of subretinal fluid (SRF). Serial changes in BCVA and CMT were analyzed by using repeated measures analysis of variance.

**Results:**

During the 6-month follow-up, the mean number of injections required and performed was 1.77 ± 0.60. The logMAR BCVA was 0.48 ± 0.26 at the baseline, 0.34 ± 0.26, 0.30 ± 0.26, 0.27 ± 0.26, 0.24 ± 0.26, and 0.23 ± 0.26 at 2-week and 1-, 2-, 3-, and 6-month follow-ups, respectively (*F* = 27.173, *P* < 0.05). CMT was 313.74 ± 144.51 *μ*m at the baseline and decreased to 263.49 ± 120.44 *μ*m, 225.91 ± 91.98 *μ*m, 195.77 ± 66.69 *μ*m, 189.74 ± 65.41 *μ*m, and 199.49 ± 81.50 *μ*m at 2-week and 1-, 2-, 3-, and 6-month follow-ups, respectively (*F* = 18.093, *P* < 0.05). Full resolution of SRF was achieved in 8 (22.9%) eyes at 1 month, 16 (45.7%) eyes at 2 months, 22 (62.9%) eyes at 3 months, and 27 (77.1%) eyes at 6 months after the initial treatment of anti-VEGF injection. No severe adverse event was noted relevant to the therapy.

**Conclusions:**

Intravitreal injection of conbercept may effectively reduce the CMT and improve the BCVA in chronic CSC in a short term of 6 months.

## 1. Introduction

Chronic central serous chorioretinopathy (CSC) commonly affects the posterior pole and is characterized by the serous detachment of the neurosensory retina and change in the retinal pigment epithelium (RPE), resulting from the leakage of RPE [1]. For most patients, this disorder is self-limited during weeks to months after the first onset. However, some patients may have difficulty in achieving full visual recovery if there is a persistent existence of subretinal fluid (SRF), serous retinal detachment, or atrophy of the RPE [2]. Therefore, the therapeutic course should be taken to minimize the chances of this happening.

Currently, CSC is treated by photodynamic therapy (PDT), laser photocoagulation, or intravitreal injection of anti-vascular endothelial growth factor (VEGF) [3–5]. Intravitreal injections of anti-VEGF, such as ranibizumab, bevacizumab, and aflibercept have been broadly used in chronic CSC [6–8]. Ranibizumab and bevacizumab are derived from a murine monoclonal antibody, while aflibercept is a recombinant fusion protein [9]. Francesco et al., Lim et al., Kim et al. and Inoue et al. all indicated that intravitreal injection of bevacizumab resulted in improvement in best-corrected visual acuity (BCVA) and anatomic structures [2, 6, 10, 11]. Bae et al. demonstrated that intravitreal ranibizumab may significantly enhance the BCVA and reduce the central macular thickness (CMT) by 6 months after the initial treatment [7]. These results showed that the intravitreal injection of anti-VEGF agents can be an effective treatment option for chronic CSC.

The clinical trials discussed above all related to monoclonal antibodies. Few studies were found in the safety and effectiveness of intravitreal injections of recombinant fusion protein for chronic CSC. Similar to aflibercept, conbercept (Lumitin, Chengdu Kanghong Biotech Co., Ltd., Sichuan, China) is a recombinant fusion protein fused by VEGF receptors one and two and the Fc portion of the human immunoglobulin G1, which can block VEGF-B, placental growth factor, and all VEGF-A isoforms [12]. Conbercept has been proven to be effective in age-related macular degeneration (AMD), central retinal vein occlusion, and other macular disorders [9, 13]. In the current study, we aim to investigate the short-term efficacy and safety of conbercept in chronic CSC. Further studies on the long-term efficacy and safety are necessary.

## 2. Patients and Methods

### 2.1. Patients

This study included 31 patients (35 eyes) diagnosed with chronic CSC who received intravitreal conbercept injection from November 2015 to May 2018 at the Hangzhou Branch of Eye Hospital of Wenzhou Medical University. Criteria for inclusion were (1) age >18 years old; (2) presence of serous detachment of neurosensory retina and RPE on optical coherence tomography (OCT) (Spectralis, Heidelberg, Germany); (3) evidence of fluorescent leakage on fundus fluorescein angiography (FFA) (Spectralis, Heidelberg, Germany); (4) abnormal appearance of dilated choriocapillaris on indocyanine green angiography (ICGA) (Spectralis, Heidelberg, Germany); (5) symptom duration ≥6 months; and (6) follow-up period ≥6 months. Criteria for exclusion were (1) secondary choroidal neovascularization; (2) accompanied with other eye diseases, such as AMD, polypoidal choroidal vasculopathy, glaucoma, or ocular trauma; (3) previous treatments, including argon laser, photodynamic therapy, or vitreoretinal surgery; and (4) history of systemic steroid usage before the onset of the clinical symptoms.

### 2.2. Methods

All the patients received intravitreal injections of conbercept (0.05 ml/0.5 mg) under sterile conditions initially. And additional injections of conbercept which were administered as needed were determined based on the BCVA and OCT findings. Such approaches were known as “1 + pro re nata (1 + PRN)” treatment regimens, namely, one intravitreal injection of conbercept at the baseline followed by as-needed reinjection. Retreatment was required if either of the following criteria was satisfied: (1) the BCVA loss was ≥0.2 logMAR; (2) evidence of persistent fluid on OCT more than a month after the previous injection [7].

Necessary ophthalmological workup was performed before treatment and at each follow-up, including slit-lamp examinations, dilated fundus examinations, intraocular pressure measurements, BCVA testing, OCT, FFA, ICGA, and other testing as needed. The BCVA was evaluated by standard logarithmic visual acuity chart and converted into logarithm of the minimum angle of resolution (logMAR) for statistical analysis. The CMT was assessed by a highly qualified ophthalmologist using manual measurement of the distance between the inner limiting membrane and RPE at the fovea on OCT.

Safety was assessed by recording systemic serious adverse events such as cardiocerebral events and any ocular adverse events such as choroidal neovascularization, vitreous hemorrhage, retinal detachment, or endophthalmitis during the study period. Follow-ups were at 2 weeks and 1, 2, 3, and 6 months after the initial injection. Observed indicators included change in BCVA and CMT and presence of SRF. All participants signed informed consents before participating in the study. And the study was conducted in accordance with the tenets of the Declaration of Helsinki and approved by the ethics committee of the hospital.

### 2.3. Statistical Analysis

Statistical analyses were performed using IBM SPSS Statistics (v 19.0; IBM Corporation, Chicago, IL). All data were expressed as means ± standard deviations. Serial changes in BCVA and CMT were compared using repeated measures analysis of variance. A *P* value of less than 0.05 was considered statistically significant.

## 3. Results

Thirty-five eyes of 31 patients with chronic CSC were enrolled. Three patients at 2 weeks, four patients at 2 months, and one patient at 3 months were lost to follow-up owing to their personal reasons. The baseline demographic data and clinical characteristics are summarized in Table 1. There were twenty-three males and eight females with a mean age of 50.66 ± 8.31 years. During the 6-month follow-up, the average number of injections required was 1.77 ± 0.60 (range, 1–3). No severe systemic and ocular adverse events were noted relevant to the therapy, such as choroidal neovascularization, vitreous hemorrhage, retinal detachment, uveitis, or endophthalmitis.

### 3.1. Change of BCVA

**Figure 1:**
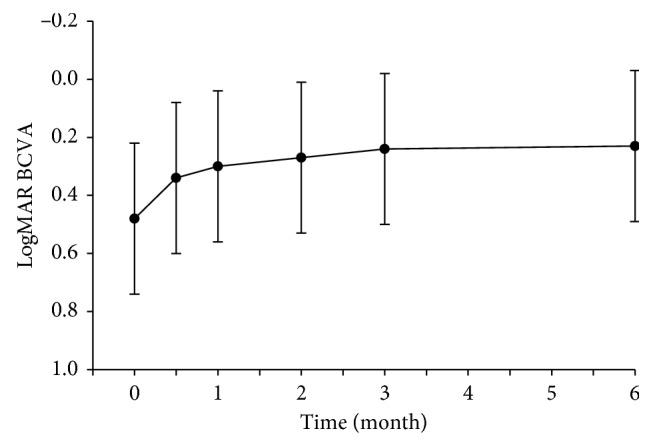
Mean logMAR BCVA ( ± SD) from baseline to month 6.

The mean logMAR BCVA was 0.48 ± 0.26 (Snellen equivalent, 20/60) at the baseline, 0.34 ± 0.26 (Snellen equivalent, 20/44) at the 2-week follow-up, 0.30 ± 0.26 (Snellen equivalent, 20/40) at the 1-month follow-up, 0.27 ± 0.26 (Snellen equivalent, 20/37) at the 2-month follow-up, 0.24 ± 0.26 (Snellen equivalent, 20/35) at the 3-month follow-up, and 0.23 ± 0.26 (Snellen equivalent, 20/34) at the 6-month follow-up. The difference between the BCVA at each follow-up and the first visit was statistically significant (*F* = 27.173, *P* < 0.05). That is, the treatment of conbercept injection improved the BCVA at each posttreatment follow-up (*P* < 0.05) (Figure 1 and Table 2).

### 3.2. Change of CMT

**Figure 2:**
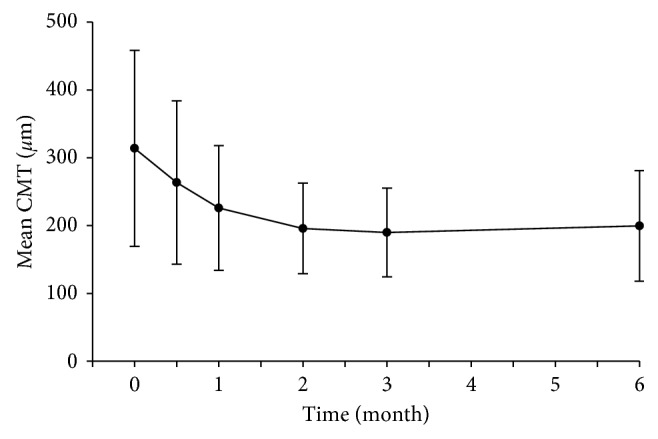
Mean CMT (±SD) from baseline to month 6.

The mean CMT was 313.74 ± 144.5 *μ*m at the baseline, 263.49 ± 120.44 *μ*m at the 2-week follow-up, 225.91 ± 91.98 *μ*m at the 1-month follow-up, 195.77 ± 66.69 *μ*m at the 2-month follow-up, 189.74 ± 65.41 *μ*m at the 3-month follow-up, and 199.49 ± 81.50 *μ*m at the 6-month follow-up. The difference between the CMT at each follow-up and the first visit was also statistically significant (*F* = 18.093, *P* < 0.05). With the initial treatment of conbercept, the mean CMT was substantially reduced at each follow-up continuously (*P* < 0.05) (Figure 2 and Table 2).

### 3.3. Chang of SRF

**Figure 3:**
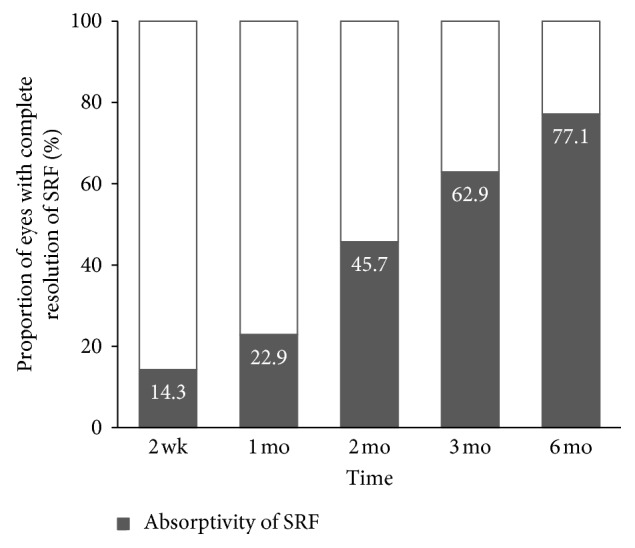
Proportion of eyes with complete resolution of SRF at each follow-up visit.

Initially, presence of SRF was found in all 35 eyes. Full resolution of fluid was achieved in 8 (22.9%) eyes at 1 month, 16 (45.7%) eyes at 2 months, 22 (62.9%) eyes at 3 months, and 27 (77.1%) eyes at 6 months after the initial treatment of anti-VEGF injection (Figure 3).

## 4. Discussion

A variety of factors were found to be associated with chronic CSC, including type A personality, stress event, elevated levels of corticosteroids, and genetic susceptibility [14]. Nonetheless, the essential pathogenesis of chronic CSC remained undiscovered with controversy. It is generally accepted that the dysfunction of RPE and the choroidal vascular hyperpermeability primarily lead to the detachment of the RPE in chronic CSC patients [15]. Therefore, some proposed on the potential efficacy in anti-VEGF treatment in chronic CSC given its antipermeability properties in decreasing the choroidal vascular hyperpermeability [16]. Currently, several kinds of anti-VEGF agents have been in use. Yun et al. demonstrated that intravitreal injection of aflibercept can significantly decrease subfoveal choroidal thickness more than ranibizumab in the treatment of neovascular AMD due to their difference in biochemical structures [17]. Since the CSC is known to be a type of pachychoroid disease associated with choroid dysfunction [18], it is a possibility that the application of intravitreal conbercept may be effective in treatment for the chronic CSC.

In our study, 33 (94%) out of 35 eyes had stable or improved vision at the time of the last follow-up. A mean reduction in CMT was found to be 114.25 *μ*m at 6 months. The study defined the complete resolution of SRF on the OCT as high responders (HRs). At the 6-month follow-up, there were 27 (77.1%) HRs without any adverse events. These results indicated that intravitreal conbercept with “1 + PRN” treatment protocol may be efficacious in resolving chronic CSC over a period of 6 months. Further study is required in determining efficacy and safety in a more frequent treatment regimen, such as monthly injection or a 3 + PRN treatment. The pathophysiology in the treatment of CSC with anti-VEGF therapy is not yet completely understood. Lim et al. discovered that the VEGF in the aqueous was significantly correlated with the symptom duration [19]. Thus, we can generally believe that anti-VEGF therapy plays a significant role in chronic CSC. Recent published studies in small case series showed that intravitreal injections of bevacizumab or ranibizumab resulted in visual improvement and CMT reduction without adverse events. Lim et al. also indicated that the bevacizumab injection may help to improve visual acuity and anatomical results [6]. Another study from Kim et al. reported 42 patients who were treated with intravitreal injections of bevacizumab had significant reduction in CMT, SRF height, and SRF volume with no visual improvement at the last follow-up. 60% of them achieved complete resolution at a mean follow-up of 8.6 months [10]. Inoue et al. reported results of one-year follow-up examinations. Their study demonstrated that intravitreal injection of bevacizumab was effective in maintaining vision and improving serous retinal detachment [11].

In this study, there are still several limitations of relatively short-term follow-up period, small sample size, lack of a control group, and fundus angiography data. Further investigations are expected in both a larger sample and a longer term of study period. The comparison between efficacy of conbercept injection and PDT may be compared to determine the most beneficial first-line treatment for chronic CSC.

## 5. Conclusion

In summary, the results of our study demonstrated significant improvement in visual acuity and anatomic structures with intravitreal injections of conbercept in chronic CSC. Intravitreal injections of conbercept may be considered as a therapeutic option to treat chronic CSC patients.

## Figures and Tables

**Table 1:** Baseline demographic and clinical characteristics of patients with chronic CSC treated with conbercept.

Parameters	
Patient number/eye	31/35
Mean age (year)	50.66 ± 8.31
Sex (male/female)	23/8
Baseline logMAR BCVA	0.48 ± 0.26
Baseline CMT (*μ*m)	313.74 ± 144.5

CSC: central serous chorioretinopathy; logMAR: logarithm of the minimum angle of resolution; BCVA: best-corrected visual acuity; CMT: central macular thickness.

**Table 2:** Mean values and standard deviations for logMAR BCVA and CMT after treatment.

	2 weeks	1 month	2 months	3 months	6 months
	*n*=32	*n*=35	*n*=31	*n*=34	*n*=35
BCVA	0.34 ± 0.26	0.30 ± 0.26	0.27 ± 0.26	0.24 ± 0.26	0.23 ± 0.26
*P* value^a^	<0.05	<0.001	<0.001	<0.001	<0.001
CMT (*μ*m)	263.49 ± 120.44	225.91 ± 91.98	195.77 ± 66.69	189.74 ± 65.41	199.49 ± 81.50
*P* value^a^	<0.05	<0.05	<0.001	<0.001	<0.05

^a^Repeated measures analysis of variance.

## Data Availability

The data used to support the findings of this study are available from the corresponding author upon request.
